# Treatment of Nocturnal Erections

**Published:** 1856-09

**Authors:** 


*Treatment of Nocturnal Erections.* By M. Debout.

When these occur during the course of a gonorrhœa, or are produced by genital erethism in the young, M. Van den Corput recommends the following pills:—Extr. of belladon. 10 centigr., fresh lupulin, camphor, *aa* 60 centig. Divide into eight pills, from one to four to be taken towards evening. The saccharate of lupulin always succeeds, when given in sufficient doses, in the relief of the erections, which are sympathetic of specific inflammation of the mucous membrane. A better combination than that of camphor and belladonna is formed with indian hemp:—Extract of indian hemp 5 centigrammes, recent lupulin 1½ grammes, sugar 9 s., for the trituration of the lupulin. To form two doses, to be taken in the evening at an hour's interval. As to the erethism of children, it is usually induced by ascarides in the rectum, and may be relieved by injections of cold water or of salt and water.—*Ibid from Ibid.*

